# 

**Published:** 1898-09-24

**Authors:** 


					<y
THE hospital. "The standard of highest parity,"?Lancet, sbpt. 24th, isas.
CANBURY'S ^ ww w. CADBURY'S
COOOA Ml: in|[ JST* ?rc COCOA
NO DRUQSIORfALKALIESlUSEB.
V
WHVCH HOSEl.TAL
No. 626. Vol. XXIV. [KSi?] Sattjbdat,
Sept. 24th, 1898.
[SubsanrtoonTornn,] pRICE TWOPENCE.

				

